# Beneficial Effect of a Multistrain Synbiotic *Prodefen^®^ Plus* on the Systemic and Vascular Alterations Associated with Metabolic Syndrome in Rats: The Role of the Neuronal Nitric Oxide Synthase and Protein Kinase A

**DOI:** 10.3390/nu12010117

**Published:** 2020-01-01

**Authors:** Pablo Llévenes, Raquel Rodrigues-Díez, Laia Cros-Brunsó, Mᵃ Isabel Prieto, Laura Casaní, Gloria Balfagón, Javier Blanco-Rivero

**Affiliations:** 1 Department of Physiology, School of Medicine, Universidad Autónoma de Madrid, Calle de Arzobispo Morcillo 4, 28029 Madrid, Spain; pablollevenes@gmail.com (P.L.); croslaia@gmail.com (L.C.-B.); gloria.balfagon@uam.es (G.B.); 2 Department of Pharmacology and Therapeutics, School of Medicine, Universidad Autónoma de Madrid, Calle de Arzobispo Morcillo 4, 28029 Madrid, Spain; raquel.rodrigues@uam.es; 3 Center for Biomedical Research Network (CIBER) in Cardiovascular Diseases, Calle de Melchor Fernández Almagro 3, 28029 Madrid, Spain; 4 Research Institute University Hospital la Paz (IdIPaz), Calle de Pedro Rico 6, 28029 Madrid, Spain; iprieto@intermic.com; 5 Department of General and Digestive Surgery, Hospital Universitario la Paz, Paseo de la Castellana 261, 28046 Madrid, Spain; 6 Research Institute of Santa Creu i Sant Pau Hospital, Carrer de Sant Quintí 77–79, 08041 Barcelona, Spain; lcasani@santpau.cat

**Keywords:** metabolic syndrome, synbiotic, hypertension, superior mesenteric artery, perivascular nitrergic innervation, nitric oxide, neuronal nitric oxide synthase, protein kinase a

## Abstract

A high fat diet (HFD) intake is crucial for the development and progression of metabolic syndrome (MtS). Increasing evidence links gut dysbiosis with the metabolic and vascular alterations associated with MtS. Here we studied the use of a combination of various probiotic strains together with a prebiotic (synbiotic) in a commercially available *Prodefen^®^ Plus*. MtS was induced by HFD (45%) in male Wistar rats. Half of the MtS animals received *Prodefen^®^ Plus* for 4 weeks. At 12 weeks, we observed an increase in body weight, together with the presence of insulin resistance, liver steatosis, hypertriglyceridemia and hypertension in MtS rats. *Prodefen^®^ Plus* supplementation did not affect the body weight gain but ameliorated all the MtS-related symptoms. Moreover, the hypertension induced by HFD is caused by a diminished both nitric oxide (NO) functional role and release probably due to a diminished neuronal nitric oxide synthase (nNOS) activation by protein kinase A (PKA) pathway. *Prodefen^®^ Plus* supplementation for 4 weeks recovered the NO function and release and the systolic blood pressure was returned to normotensive values as a result. Overall, supplementation with *Prodefen^®^ Plus* could be considered an interesting non-pharmacological approach in MtS.

## 1. Introduction

Obesity affected two billion people worldwide in 2015 with annual costs reaching two trillion USD [1]. The global prevalence has increased dramatically in the last four decades, reaching 10.8% in adult men and 14.9% in adult women [2]. Chronic consumption of a high-fat diet (HFD) induces overweight and is the main trigger for the development of metabolic syndrome (MtS) characterized by dyslipidemia, hypertension and impaired glucose homeostasis [3,4]. MtS is associated with high mortality and morbidity due to an increase in the prevalence of cardiovascular diseases, diabetes, chronic kidney diseases, cancer and musculoskeletal diseases [2].

The accumulation of adipose tissue promotes a pro-inflammatory and pro-oxidative microenvironment, leading to a chronic low-grade inflammation state [5]. Moreover, the secretion of adipokines from the adipose tissue alters the function of multiple vasoactive factors, increases peripheral vascular resistance and leads to hypertension. Evidence indicates that the blood flow and tissue perfusion required for cardiovascular system homeostasis is affected by nitric oxide (NO) synthesis [6,7]. Other factor implicated in the regulation of vascular resistance is perivascular innervation, specifically in certain vessels such as the mesenteric vascular bed, where blood flow is approximately 20–30% of the total cardiac output [8]. Previously we have reported that the neural control of mesenteric vasomotor tone is altered in rats fed on HFD for a short term, partly due to a decrease in neuronal NO release from perivascular nitrergic innervation [9,10].

Increasing evidence links the development of MtS and chronic low-grade inflammation to dysbiosis, an imbalance in the intestinal flora [11,12,13]. Some studies demonstrated that dysbiosis promotes the development of low-grade inflammation and consequently MtS, and others that dysbiosis is the result of low-grade inflammation during obesity and MtS. Either way, the modulation of dysbiosis by dietary strategies based on alterations of gut microbiome improves the health outcome of MtS [14]. The supplementation of probiotics, live bacterial strains, have been shown to modulate the alteration of gut microbiome [15] and to improve MtS symptoms, such as insulin resistance, lipid profile and high blood pressure in animal models [16,17].

It is considered that multistrain and/or multi-species probiotics have been shown in animal models to be more effective than monostrain probiotics [18]. Additionally, the use of prebiotics, non-digestible oligosaccharides that stimulates the growth of desired bacteria, together with a probiotic is more effective than probiotics alone by improving survival and implantation of live microbes in the gastrointestinal tract [19]. The commercial synbiotic formulation *Prodefen^®^ Plus* combines various probiotic strains (*Lactobacillus rhamnosus*, *Lactobacillus casei*, *Lactobacillus acidophilus*, *Lactobacillus bulgaricus*, *Streptococcus thermophilus*, *Bifidobacterium breve* and *Bifidobacterium infantis*) together with a prebiotic, fructooligosaccharides. Both *Prodefen^®^ Plus*, and a similar synbiotic commercial formula *Prodefen^®^* exert a beneficial effect in antibiotic-associated diarrhoea and acute gastroenteritis in children, respectively [20,21]. Regarding MtS, *Prodefen^®^* has shown an antihypertensive effect in spontaneously hypertensive rats [22]. Nevertheless, the possible beneficial effect of *Prodefen^®^ Plus* in MtS remains unknown.

Given the above, we hypothesized that a supplementation with the commercially available synbiotic formula *Prodefen^®^ Plus* might improve the cardiometabolic alterations related to MtS, leading to a reduction in the administration of any pharmacological treatment. Hence, in the present study we aim to assess the effect of *Prodefen^®^ Plus* in rats fed on HFD that develop MtS, with a special focus on the understanding of the vascular mechanisms implicated in the development of hypertension.

## 2. Materials and Methods

### 2.1. Animals and Diet

All experimental procedures were approved by the Ethical Committee of the Universidad Autónoma de Madrid, and the Comunidad de Madrid (PROEX 322/16), are in compliance with NIH guidelines and follow the European Parliament Directive 2010/63/EU guidelines. Twenty-four male Wistar rats (initial weight 209.5 ± 3.1 g) were purchased from Harlan Ibérica SL, Barcelona, Spain and housed in the Animal Facility of the Universidad Autónoma de Madrid (Registration number EX-021U). Rats were held in groups of 2 in appropriate cages in controlled environmental conditions (20–24 °C, 55% relative humidity and 12 h light-dark cycle). All rats had access to drinking water and specific rat chow *ad libitum*.

Animals were randomly divided into three groups: (I) Rats fed a commercial standard diet (4.0% fat; 2.9 Kcal/g; Tekland global rodent diet 2014, ENVIGO, Huntingdon, UK) for 12 weeks as a control group (CT; *n* = 8); (II) rats with metabolic syndrome (MtS, *n* = 8), fed a HFD (45% fat; 4.6 Kcal/g; 58V8, TestDiet, Columbia, USA) for 12 weeks; and (III) rats with MtS, fed a HFD (45% fat, 4.6 Kcal/g) for 12 weeks, supplemented with the multistrain synbiotic *Prodefen^®^ Plus* (adjusted doses to 10^7^ colony forming units (c.f.u.)/day) for the last 4 weeks (MtS-SYNB, *n* = 8).

**Figure 1:**
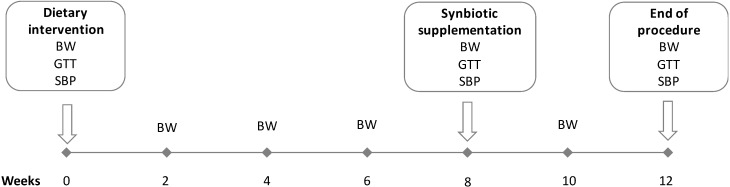
Diagrammatic representation of the 12-weeks experimental procedure. Abbreviations: body weight (BW), glucose tolerance test (GTT), systolic blood pressure (SBP).

*Prodefen^®^ Plus* (formulation which combines 990 mg of fructooligosaccharides with various probiotic strains −10^10^ c.f.u. *Lactobacillus rhamnosus* LGG and 10^9^ c.f.u. of a mixture of: *Lactobacillus casei, Streptococcus thermophilus, Bifidobacterium breve, Lactobacillus acidophilus, Bifidobacterium infantis and Lactobacillus bulgaricus*), was generously provided by Italfarmaco S.A. This synbiotic formula was administered to the rats dissolved in drinking water, once the obesity had been stablished [9,10]. The dose (10^7^ c.f.u./day) and administration time (4 weeks) of *Prodefen^®^ Plus* was chosen based on preliminary pilot studies, choosing the lowest dose/time in which we found a systemic effect. The body weight was measured every two weeks. During the last 4 weeks, the water intake was measured every two days to assure the intake of appropriate dose of the synbiotic. The summary of the experimental procedure is represented in Figure 1.

#### 2.1.1. Glucose Tolerance Test

The oral glucose tolerance test (GTT) was performed at 0, 8 and 12 weeks according to a standard protocol [23,24]. After an overnight fasting, a single oral dose (2 g/kg of body weight) of glucose was delivered. Blood glucose was then measured from the tail vein just before, and at 15, 30, 60, 90 and 120 min after glucose intake, using test strips and reader (FreeStyle Optium^®^, Abbot Laboratories S.A., Madrid, Spain) (Figure 1).

#### 2.1.2. Systolic Blood Pressure

The systolic blood pressure was measured at 0, 8 and 12 weeks in awake rats by a tail-cuff method, using a caudal artery plethysmograph (NIPREM 645, Cibertec S.A., Madrid, Spain) as published previously [9]. (Figure 1)

### 2.2. Animal Euthanasia, Sample Collection and Sample Analysis

After an overnight fasting, rats were anesthetized (100 mg/kg ketamine hydrochloride and 12 mg/kg Xylazine; i.m.) and euthanized by exsanguination by the infrahepatic inferior cava vein puncture. Visceral and epididymal white adipose pads were collected for posterior dissection. Left tibia length was measured as a parameter to evaluate body weight.

#### 2.2.1. Liver Histology

Liver was extracted, rinsed in a saline solution and weighed. The left lateral liver lobe was cryoprotected (30% *w*/*v* sucrose in phosphate saline buffer) and frozen at −70 °C until use. Liver samples were embedded in optimum cutting temperature compound (OCT Tissue Tek, Sigma-Aldrich, Madrid, Spain), 5 μm cryostat sections were stained with lipid dye Sudan III and random images were taken at 20× magnification. Quantification was performed using Image-Pro Plus 7 software (Media Cybernetics, MD, USA).

#### 2.2.2. Serum Biochemical Parameters

Blood samples were kept at room temperature for 2 h and were centrifuged afterwards (2000× *g* 15 min, 4 °C). The serum was then collected and kept at −70 °C until use. Serum concentration of total cholesterol (TC), triglycerides (TG) and high density lipoproteins (HDL) were measured by biochemical analyser RAL LC5 (Barcelona, Spain). Low density lipoprotein (LDL) levels were indirectly calculated using the Friedewald formula: LDL = TC-((TG/5) + HDL) as published previously [25].

#### 2.2.3. Vascular Reactivity

The superior mesenteric artery was carefully dissected, cleaned of connective tissue and maintained at 4 °C in Krebs-Henseleit solution (in mmol/L: 115 NaCl, 25 NaHCO_3_, 4.7 KCl, 1.2 MgSO_4_.7H_2_O, 2.5 CaCl_2_, 1.2 KH_2_PO_4_, 11.1 glucose, and 0.01 Na_2_EDTA) bubbled with a 95% O_2_ 5% CO_2_ mixture until use. The segments were endothelium-denuded by gently rubbing the luminal surface of the segments with a thin wooden stick to eliminate the main source of vasoactive substances. Some segments were quickly frozen in liquid nitrogen and maintained at −70 °C.

Vascular reactivity experiments were performed in endothelium denuded segments of 2 mm length according to previously published method [26]. Briefly, two parallel stainless-steel pins were introduced through the lumen of the vascular segment: one was fixed to the bath wall and the other connected to a force transducer (Grass FTO3C; Quincy, MA, USA) that was connected to a model 7D Grass polygraph. For electric field stimulation (EFS) experiments, segments were mounted between two platinum electrodes 0.5 cm apart and connected to a stimulator (Grass, model S44) modified to supply the appropriate current strength. Segments were suspended in an organ bath containing 5 mL of KHS at 37 °C continuously bubbled with a 95% O_2_ 5% CO_2_ mixture (pH 7.4). The segments were subjected to a tension of 4.9 mN, which was readjusted every 15 min during a 90 min equilibration period before drug administration. After this, the vessels were exposed to 75 mmol/L KCl to confirm their functional integrity. The absence of vascular endothelium after the washout period was tested by the inability of 10 µmol/L acetylcholine (ACh) to relax segments precontracted with noradrenaline. Endothelium removal did not alter the contractions elicited by KCl [10,23].

Frequency-response curves to EFS (1, 2, 4, 8 and 16 Hz) were performed. The parameters used for EFS were 200 mA, 0.3 ms, 1–16 Hz, for 30 s with an interval of 1 min between each stimulus, the time required to recover the basal tone. A washout period of at least 1 h was necessary to avoid desensitisation between consecutive curves. Two successive frequency-response curves separated by 1 h intervals produced similar contractile responses [26].

To analyse the participation of NO in the EFS-induced response in our experimental procedure, 0.1 mmol/L Nω-nitro-L-arginine methyl ester (L-NAME), a non-specific inhibitor of nitric oxide synthase, was added to the bath 30 min before performing the second frequency–response curve. The vasodilator response to the NO donor, diethylamine NONOate, (DEA-NO, 0.1 nmol/L–0.1 mmol/L was determined in noradrenaline-precontracted segments from all experimental groups.

#### 2.2.4. Nitric Oxide Release

Nitric oxide (NO) release experiments were performed in endothelium-denuded mesenteric segments using the fluorescence probe 4,5-diaminofluorescein (DAF-2) as previously described [9,10,27]. Briefly, segments from all the experimental groups were subjected to an equilibration period of 60 min in HEPES buffer (in mmol/L: NaCl 119; HEPES 20; CaCl_2_ 1.2; KCl 4.6; MgSO_4_ 1; KH_2_PO_4_ 0.4; NaHCO_3_ 5; glucose 5.5; Na_2_HPO_4_ 0.15; pH 7.4) at 37 °C. Afterwards, arteries were transferred to a HEPES-filled 400 μL chamber and incubated with 2 µmol/L DAF-2 for 30 min. Then the medium was collected to measure basal NO release. Once the organ bath was refilled, cumulative EFS pulses of 30 s (1, 2, 4, 8 and 16 Hz) were applied at 1 min intervals. The fluorescence of the medium was measured at room temperature using a spectrofluorometer (Jenway 6280 Fluorimeter) with excitation wavelength set at 492 nm and emission wavelength at 515 nm. Some segments were incubated with 1 μmol/L H89 (a PKA (protein kinase A) inhibitor), 0.1 μmol/L calfostin C (a PKC (protein kinase C) inhibitor), or 10 μmol/L LY 294002 (a phosphatidylinositol 3-kinase -PI3K- inhibitor). The EFS-induced NO release was calculated by subtracting basal NO release from that evoked by EFS. Furthermore, blank samples were collected in the same way from segment-free medium in order to subtract background emission. The amount of NO released was expressed as arbitrary fluorescence units (A.F.U.)/mg tissue.

#### 2.2.5. PKA and PKC Activity Assays

PKA and PKC activities were determined using a PKA and PKC kinase activity assay kit (Abcam, Cambridge, UK), respectively following the manufacturers’ protocols. Briefly, frozen arteries from all experimental groups were homogenised in a lysis buffer (0.01 mmol/L Tris-HCl, 1 mmol/L sodium vanadate, 1% SDS, pH 7.4), and centrifuged at 12,000× *g* for 10 min at 4 °C. The supernatant was then collected and used for the assay. Protein content was measured using a DC protein assay kit (BioRad, Madrid, Spain). Results were expressed as optical density (O.D.) units/μg protein.

#### 2.2.6. Western Blot

Western blot analysis was performed as previously described [8,9]. Briefly, arteries were homogenized, and 30 μg protein was loaded in each lane. A mouse monoclonal antibody against neuronal nitric oxide synthase (nNOS, 1:2000, BD Biosciences), a rabbit polyclonal anti-pan-AKT antibody (1:1000, Abcam), or a rabbit polyclonal anti-pan-AKT (phospho T308, 1:500, Abcam) were used. The development and quantification of the images were performed using Quantity One software (Windows v4.6.6, Bio-Rad, Madrid, Spain). The same membrane was used to correct protein expression in each sample, by means of a monoclonal anti-β-actin−peroxidase antibody (1:50,000; Sigma-Aldrich, Madrid, Spain).

#### 2.2.7. Detection of Superoxide Anions

Superoxide anion levels were measured using lucigenin chemiluminescence, as previously described [10,27]. Briefly, endothelium-denuded segments from all experimental groups were equilibrated for 30 min in HEPES buffer at 37 °C, transferred to test tubes that contained 1 mL HEPES buffer (pH 7.4) with lucigenin (5 µmol/L) and then kept at 37 °C. The luminometer (Optocom I, GEM Biomedical Inc., Hamden, CT, USA) was set to report arbitrary units of emitted light; repeated measurements were collected (10 s intervals for 5 min) and averaged. 4,5-Dihydroxy-1,3-benzene-disulphonic acid “Tiron” (10 mmol/L), a cell-permeant, non-enzymatic superoxide anion scavenger, was added to quench the superoxide anion-dependent chemiluminescence. Blank samples (HEPES without arterial segment) were collected in the same way from culture medium without mesenteric segments to subtract background emission.

### 2.3. Drugs Used

L-Noradrenaline hydrochloride, ACh chloride, diethylamine NONOate diethylammonium salt, Nω-nitro-L-arginine methyl ester (L-NAME) hydrochloride, lucigenin, tiron, DAF-2, calfostin C, H89, and LY294002 were used. All drugs were purchased from Sigma-Aldrich (Spain) except for LY294002 and H89, which were obtained from Tocris (Spain). Stock solutions (10 mmol/L) of drugs were made in distilled water, except for NA, which was dissolved in a NaCl (0.9%)–ascorbic acid (0.01% *w*/*v*) solution, or DAF, H89 and calfostin C and LY294002, which were dissolved in dimethyl sulfoxide.

### 2.4. Data Analysis

Graph representation and statistical analysis were performed using GraphPad Prism 6.0 software (CA, USA). The adipose tissue pads and liver weight were normalised using tibia length. The responses induced by EFS were expressed as a % of the initial contraction elicited by 75 mmol/L KCl. To compare the effect of preincubation with L-NAME in EFS-induced contraction experiments, differences between areas under the curve (dAUC) were analysed. The relaxation induced by DEA-NO was expressed as a % of the initial contraction elicited by NA. Results were expressed as mean ± S.E.M. The body weight evolution, the glucose tolerance test and the vasomotor responses to DEA-NO or EFS were compared by means of an unpaired two-way analysis of variance (ANOVA). When comparing the effect of L-NAME on the EFS-induced contraction, a paired two-way ANOVA was used. For the body weight gain, food and water intake, lipid profile, blood pressure, KCl, dAUC, NO, superoxide anion, PKA activity, PKC activity and Western blot densitometry analyses, a Shapiro-Wilk test was applied to confirm the normality of the population data, followed by a one-way ANOVA with a Newman–Keuls *post-hoc* test. *p* < 0.05 was considered significant.

## 3. Results

### 3.1. The Effect of HFD and Synbiotic Supplementation on the Body Weight Gain and Lipid Profile

**Figure 2:**
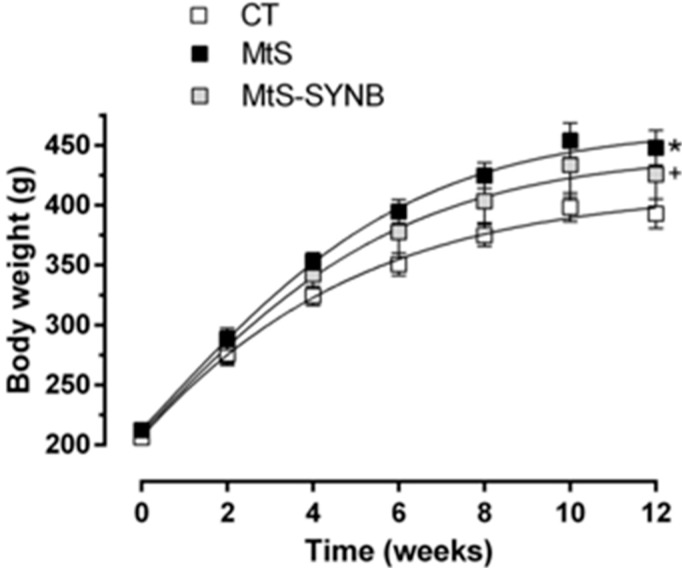
Body weight evolution in CT (control group), MtS (metabolic syndrome) and MtS-SYNB (MtS rats supplemented with *Prodefen® Plus*) groups over 12 weeks. Body weight is expressed as mean ± S.E.M. Statistical analysis: Unpaired two-way ANOVA. * *p* < 0.05 CT vs. MtS; + *p* < 0.05 CT vs. MtS-SYNB. *n* = eight animals per group.

In the present study we induced obesity in Wistar rats by the administration of a HFD (45% fat). The obesity was established after 8 weeks. At week eight, eight rats out of 16 fed on a HFD were administered a commercial synbiotic supplement *Prodefen^®^ Plus*. After 12 weeks, the body weight increase was significantly higher in MtS rats compared to CT. The supplementation of *Prodefen^®^ Plus* did not affect the body weight in MtS rats (MtS-SYNB) (Figure 2).

The body weight increase in MtS and MtS-SYNB group was accompanied by an increase in epididymal and visceral adipose pads. The food intake in MtS and MtS-SYNB group was lower and conversely the calories intake was higher. Water intake was comparable among groups (Table 1).

**Figure 3:**
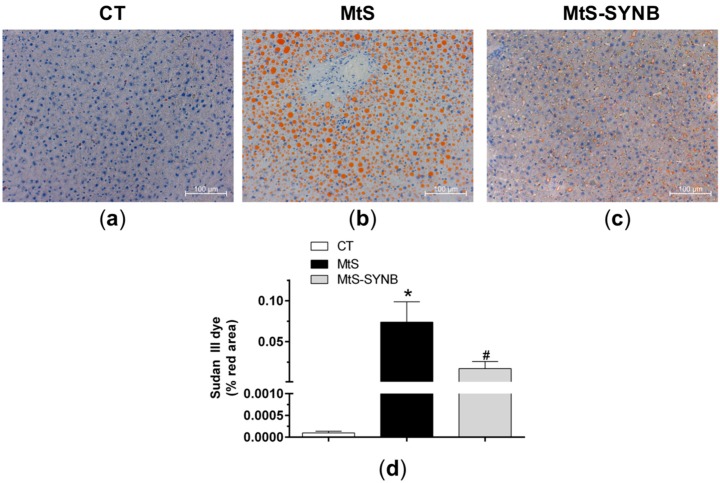
Liver sections were stained with Sudan III lipid dye. Representative images (20 × magnification) from CT (**a**), MtS (**b**) and MtS-SYNB (**c**) rats are shown. The scale bar represents 100 µm. (**d**) Quantitative analysis of Sudan III lipid dye staining. Results are represented as % of stained area (mean ± S.E.M). Statistical analysis: One-way ANOVA followed by a Newman–Keuls *post-hoc* test. * *p* < 0.05 CT vs. MtS; # *p* < 0.05 MtS vs. MtS-SYNB.

Total cholesterol, LDL and HLD levels did not differ between groups, while the levels of plasma TG were significantly increased in the MtS group as compared to CT group and were returned to control levels in MtS-SYNB group (Table 1). Given the fact that increased TG levels leads to a development of liver steatosis, we next analysed the presence of lipids in liver sections by Sudan III lipid dye. Even though liver weight was comparable among groups, the HFD led to a development of steatosis as demonstrated by the presence of lipid vacuoles throughout the hepatic left lateral lobe. Interestingly, supplementation of *Prodefen^®^ Plus* decreased the level of steatosis, even though not to the control state (Figure 3).

### 3.2. The Effect of Synbiotic Supplementation on MtS Induced Alterations

**Figure 4:**
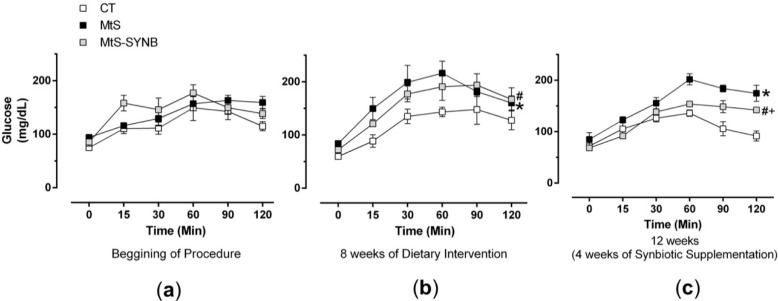
Evolution of insulin resistance measured by glucose tolerance test in CT, MtS and MtS-SYNB rats at the beginning of procedure (**a**), after 8 weeks of diet intervention (**b**) and after 12 weeks once the synbiotic supplementation terminated (**c**). Results are expressed as mg glucose/dL (mean ± S.E.M). Statistical analysis: Unpaired two-way ANOVA. * *p* < 0.05 CT vs. MtS; # *p* < 0.05 MtS vs. MtS-SYNB; + *p* < 0.05 CT vs. MtS-SYNB.

MtS is related to several metabolic alterations, therefore next we analysed the effect of HFD and synbiotic supplementation on glucose homeostasis. Although basal glycemia was comparable among groups, insulin resistance was developed already at week eight of HFD and was maintained until week 12. The insulin resistance was ameliorated by *Prodefen^®^ Plus* supplementation, even though not to the control state (Figure 4).

Hypertension is also related to MtS, therefore next we measured the systolic blood pressure using tail cuff method. At week 0 all the animals showed a normotensive phenotype. At week eight, animals fed on a HFD showed an increased systolic blood pressure reaching levels considered as hypertension. The supplementation of *Prodefen^®^ Plus* for 4 weeks normalized the systolic blood pressure reaching levels comparable to the control group (Table 2).

### 3.3. Mechanism of HFD-Induced Hypertension

#### 3.3.1. Nitric Oxide Release

The alterations in blood pressure are partially linked to modifications in peripheral vascular resistance. Among the multiple vasoactive factors which regulate vascular tone in superior mesenteric artery, NO has a relevant role, acting as a potent vasodilator. Given the great relevance of nitrergic innervation in the regulation of mesenteric resistance, we analysed the release of neuronal NO release in segments from all experimental groups. The application of an EFS pattern induced NO release in mesenteric segments without endothelium from all groups. As expected, NO release was significantly decreased in MtS group as compared to CT group. Interestingly, the supplementation with *Prodefen^®^ Plus* for 4 weeks recovered the NO release levels comparable to the control group (Table 3).

#### 3.3.2. Functional Role for Neuronal Nitric Oxide: Vascular Function

**Figure 5:**
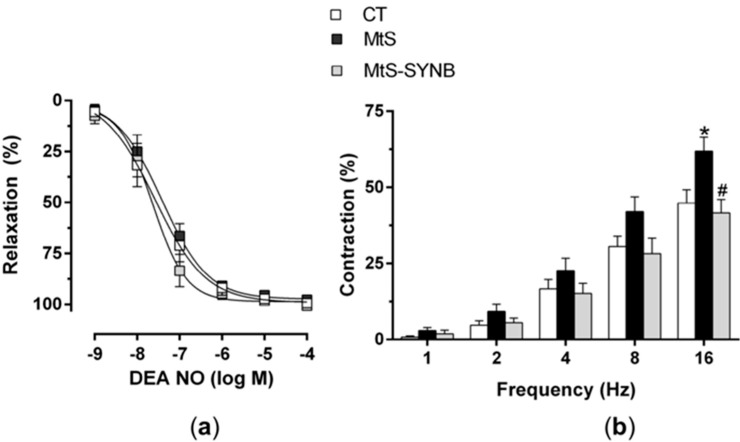
(**a**) Vasodilator response to DEA-NO in mesenteric segments from CT, MtS and MtS-SYNB rats. Results are expressed as % of relaxation elicited by NA (in mN: CT: 11.81 ± 1.1; MtS: 12.36 ± 1.03; MtS-SYNB: 14.41 ± 1.28). Mean ± S.E.M. (**b**) Vasoconstrictor response to EFS in mesenteric segments from CT, MtS and MtS-SYNB rats. Results are expressed as a % of previous contraction elicited by KCI (in mN: CT: 13.03 ± 0.74; MtS: 11.55 ± 0.51; MtS-SYNB: 12.45 ± 0.72). Mean ± S.E.M. Statistical analysis: Unpaired two-way ANOVA. * *p* < 0.05 CT vs. MtS; ^#^ *p* < 0.05 MtS vs. MtS-SYNB.

Alteration in blood pressure is linked to modifications in smooth muscle sensitivity to nitric oxide (NO). However, in our model we did not find any differences in the vasodilator response to NO donor DEA-NO among groups (Figure 5a).

**Figure 6:**
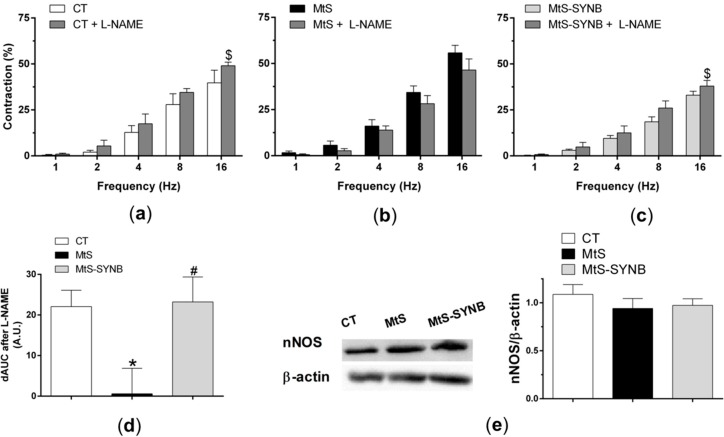
Effect of preincubation with 0.1 mmol/L L-NAME (non-specific NOS inhibitor) on the vasoconstrictor response elicited by EFS in mesenteric segments from CT (**a**), MtS (**b**) and MtS-SYNB (**c**) rats. Results (mean ± S.E.M.) are expressed as a % of previous contraction elicited by KCl. Statistical analysis: Paired two-way ANOVA. $ *p* < 0.05 segments without L-NAME vs. segments with L-NAME. (**d**) Differences of area under the curve (dAUC) in the absence or presence of 0.1 mmol/L L-NAME. Statistical analysis: One-way ANOVA followed by a Newman–Keuls *post-hoc* test. * *p* < 0.05 CT vs. MtS; # *p* < 0.05 MtS vs. MtS-SYNB. (**e**) Expression of nNOS in mesenteric segments from CT, MtS and MtS-SYNB rats. The blot is representative of eight separate segments from each group. The graph on the right shows densitometric analyses of nNOS expression. Results (mean ± S.E.M.) are expressed as the ratio of the signal obtained for nNOS and that obtained for β-actin.

Next, we aimed to determine whether the observed alteration in NO release have a relevant functional role. We applied EFS to induce a frequency-dependent contractile response in endothelium-denuded mesenteric segments. An enhanced vasoconstriction response was observed in MtS group, and the supplementation with *Prodefen^®^ Plus* for 4 weeks diminished this vasoconstriction response (Figure 5b). We next inhibited the NO synthesis with an unspecific NO synthase (NOS) inhibitor L-NAME (0.1 mmol/L) and observed a potentiation in the vasoconstriction response in CT and MtS-SYNB groups, while it exerted no effect in arteries from animals from MtS group (Figure 6a–d).

#### 3.3.3. Mechanisms Implicated in Neuronal Nitric Oxide Release

Alterations in nNOS expression and/or activity can be the responsible for the differences in NO release observed. We found that the expression of nNOS was comparable among groups (Figure 6e).

**Figure 7:**
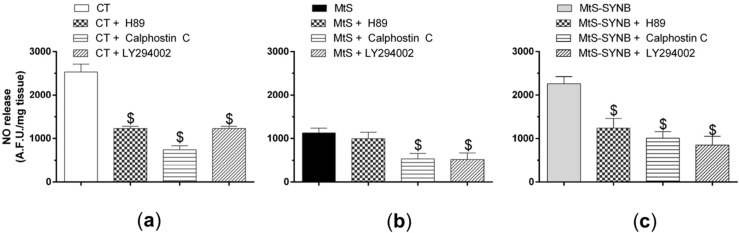
Mesenteric segments from CT (**a**), MtS (**b**) and MtS-SYNB (**c**) rats were preincubated with a protein kinase A –PKA- inhibitor H89 (1 µmol/L), a protein kinase C –PKC- inhibitor Calfostin C (0.1 µmol/L) or a phosphatidylinositol 3-kinase -PI3K- inhibitor LY294002 (10 µmol/L). NO release was induced by EFS. Data are expressed as arbitrary fluorescence units (A.F.U.)/mg tissue. Statistical analysis: One-way ANOVA followed by a Newman–Keuls *post-hoc* test. $ *p* < 0.05 conditions without inhibitor vs. conditions with respective inhibitor.

PKA, PKC and PI3K/AKT signalling pathways play a crucial role in the activation of nNOS. To analyze the involvement of the PKA pathway, we preincubated the endothelium-denuded mesenteric segments with a PKA inhibitor H89 (1 µmol/L) before EFS stimulation and measurement of NO release. We observed that H89 diminished EFS-induced NO release similarly in the CT group (60.69 ± 3.45% of inhibition) and in MtS-SYNB group (50.19 ± 13.95% of inhibition) while it produced a lower effect in MtS group (20.48 ± 11.79% of inhibition). We next analysed the involvement of the PKC pathway by using a PKC inhibitor Calfostin C (0.1 µmol/L), findig a similar inhibition by in EFS-induced NO release among the experimental groups (% of inhibition: CT: 76.41 ± 6.53; MtS: 57.33 ± 22.64; MtS-SYNB: 57.54 ± 12.71). Similar results were found after preincubation with LY394002 (10 µmol/L), a PI3K inhibitor (% of inhibition: CT: 83.96 ± 8.61; MtS: 60.51 ± 10.06; MtS-SYNB: 64.22 ± 8.46) (Figure 7a–c).

**Figure 8:**
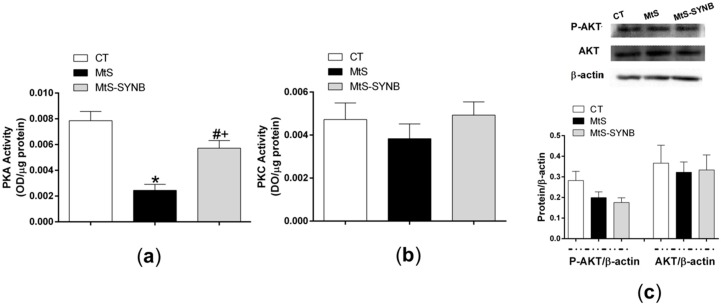
(**a**) PKA activity, and (**b**) PKC activity in mesenteric arteries from CT, MtS and MtS-SYNB rats. Results are represented as optical density (OD) units/µg protein (mean ± S.E.M). Statistical analysis: One-way ANOVA followed by a Newman–Keuls *post-hoc* test. * *p* < 0.05 CT vs. MtS; # *p* < 0.05 MtS vs. MtS-SYNB; + *p* < 0.05 CT vs. MtS-SYNB. (**c**) Western blot analysis for total AKT (AKT) and phosphorylated AKT at the T308 residue (P-AKT) in mesenteric arteries from CT, MtS and MtS-SYNB rats. Each lane is representative of eight isolated arterial segments from different animals in each group. Lower graph shows densitometric analyses of the protein expression. Results (mean ± S.E.M) are expressed as protein expression relative to β-actin expression.

This finding coincides with the PKA activity, which was significantly reduced in the MtS group as compared to the other two groups (Figure 8a). Similarly to previous findings, PKC activity and AKT expression and phosphorylation were comparable among groups (Figure 8b,c).

#### 3.3.4. Neuronal Nitric Oxide Bioavailability: Oxidative Stress

Although oxidative stress can modulate NO function by diminishing its bioavailability, we observed that superoxide anion release was similar among groups (Table 3).

## 4. Discussion

Dietary factors, especially hypercaloric diets, are the main contributors to the aetiology of MtS [28]. Chronic consumption of HFD induces overweight along with metabolic and cardiovascular alterations, such as insulin resistance, dyslipidemia or hypertension, among others, being all of these symptoms of the MtS [3,4]. Interestingly, studies in animal models demonstrated that HFD intake is related to gut dysbiosis [29,30]. Gut dysbiosis can develop as early as 2 weeks on HFD, long before the development of MtS-related symptoms [31].

Diets with a 45–60% of the energy derived from fats have been reported to promote a great weight gain in animal models [32,33]. Here we have induced obesity by a diet containing 45% fat and consistently with previous studies, we observed an increase in caloric intake together with body weight gain coinciding with an increased weight of both visceral and epididymal adipose pads [9,10]. Furthermore, in our study, the supplementation of *Prodefen^®^ Plus* for 4 weeks did not alter the body weight nor the adiposity. Inconsistent results regarding the effect of a synbiotic supplementation on body weight or adiposity index have been reported, showing either improvements [34,35] or no alterations in the weight gain [36,37]. The discrepancies observed are probably caused by different experimental procedures including the use of various probiotic strains. Clinical trials have also reported inconsistent results in body weight changes after a probiotic or synbiotic supplementation [38], moreover clinical trials also usually included changes in life or nutritional habits [39]. Therefore, more studies are needed to evaluate the role of synbiotic supplementation on body weight change.

In our study, the level of TG was increased in rats fed with HFD as compared to standard diet, while TC, HLD and LDL were not, similarly to previously published studies [9], but contrasting with others, in which the dietary intervention was different from ours [24,40]. Liver steatosis is linked to an increase in either TG and/or cholesterol levels, therefore in our study the liver steatosis observed in rats fed with HFD was caused by an increase in TG levels, which is comparable to other studies [40,41]. The supplementation of a synbiotic in our study decreased the TG levels to those achieved with a standard diet together with the diminishment of hepatic lipid deposition as previously described [41,42]. Even though basal glycemia was not altered in the MtS group, an insulin resistance was established already at week eight and was improved by synbiotic administration. A number of studies demonstrated that synbiotic supplementation leads to amelioration or a complete recovery of glucose homeostasis [34,36,37], that can be linked to a normalization in adipokine levels [41,43]. Furthermore, the lowering of plasma TGs levels can also lead to the insulin resistance improvement [44].

The adipokines secreted by the adipose tissue alter the function of multiple vasoactive factors participating in the increase in the peripheral vascular resistance and consequently in the development of MtS-linked hypertension [5]. The HFD in rodents is associated with an increase or no modification of blood pressure depending on the diet composition and duration [9,36,45]. Here we observed that HFD induced an increase in systolic blood pressure, reaching levels that are considered as hypertension. Synbiotic supplementation for 4 weeks diminished systolic blood pressure to values considered as normotensive. The release of antihypertensive factors, such as propionate or butyrate, by the probiotic strains, as well as the normalization of adipokine levels might explain the decrease in blood pressure [41,43,46].

Previous works from our group have demonstrated that the mesenteric vasculature, especially superior mesenteric artery, plays a pivotal role in the maintenance of peripheral resistance through the release of vasodilators, such as NO [8]. Although alterations in blood pressure have been linked with modifications in smooth muscle sensitivity to NO [6,47], we found no differences in the vasodilator response to NO donor DEA-NO among our experimental groups, allowing us to rule out this possibility.

NO in the vascular tissue is predominantly released from the endothelium, and it has been demonstrated that the decrease in endothelial NO release in obesity and MtS is the main factor for endothelial dysfunction [3,48]. Aside from endothelium, NO is also released from perivascular nitrergic innervation and this release is altered in situations in which vascular resistance is decreased [47,49]. Previously we have demonstrated that HFD intake diminishes NO release in superior mesenteric artery [9,10]. Similarly to observed with endothelial NO [16,36], here we have shown that the supplementation of MtS rats with synbiotics restore neuronal NO release, reaching levels comparable in the CT group. NO released from nitrergic nerve terminals is able to decrease in a great extent the maximal arterial tone elicited by the sympathetic neurotransmitter noradrenaline [23,50]. In situations where the vascular resistance is increased, we have previously observed either a potentiation in nitrergic function, which aims to compensate the greater vascular resistance [23,51], or a blunted nitrergic role, thereby participating in the enhancement of peripheral vascular resistance a consequently, in the development of hypertension [47,49]. Here we observed an enhanced EFS-induced vasoconstrictor response in MtS rats, as expected [9,10], and that the supplementation with *Prodefen^®^ Plus* diminished this vasoconstriction. When inhibiting NO synthesis with the unspecific NOS inhibitor L-NAME, we observed a potentiation in the vasoconstriction in segments from CT and MtS-SYNB rats, while it exerted no effect in arteries from MTS animals, as expected [9,10]. Consequently, we can confirm that the supplementation with *Prodefen^®^ Plus* restored the nitrergic function lost by MtS, therefore improving the vascular resistance in this pathology.

Next, we wanted to find out whether the expression or the activity of nNOS, the enzyme responsible for the synthesis of NO, is affected by MtS and synbiotic supplementation. We and others have reported that the decrease in the different NOS isozymes, in different tissues is related to obesity and MtS [9,10,52,53,54] and that probiotic or synbiotic supplementation led to an increase or no modification in the expression of these isoforms [16,55,56]. In the present study we did not observe any alteration in the nNOS expression among groups, a surprising finding given the fact that previously we observed a diminishment of nNOS expression in superior mesenteric artery in rats on a HFD [9,10]. Therefore, we assumed that the increased fat content of the diet and prolonged administration activated compensatory mechanisms, which might explain this difference. Consequently, we assumed that the differential NO released among groups might be due to an alteration in nNOS activation rather than nNOS expression, as we found in spontaneously hypertensive rats supplemented with the similar formula *Prodefen^®^* [22].

Different kinases, such as PKA, PKC or PI3K/AKT pathways are essential for multiple physiological responses. Alterations in these pathways have been reported in different MtS models, including increases, decreases and no modifications in their activities [53,57,58,59]. Even though only few inconsistent results were reported regarding the effect of probiotics and synbiotics on those pathways [60,61,62,63,64,65,66], their role in the phosphorylation and subsequent activation of nNOS is well demonstrated [27,51,67,68]. We found that the inhibition of either PKC or PI3K with calfostin C or LY294002 respectively reduced EFS-induced NO release in a similar extent in the three experimental groups. These results suggest that neither MTS nor supplementation with *Prodefen^®^ Plus* altered the participation of PKC or PI3K/AKT signalling pathways in our experimental conditions. This result agrees with the fact that both the PKC activity, and the expression/activation of AKT were similar in the three experimental groups. Concerning PKA, the preincubation with its specific inhibitor H89 blunted EFS-induced NO release similarly in both CT and MtS-SYNB rats, while it exerted no effect in arteries from MtS animals. This result correlates with the diminished PKA activity observed in segments from MtS rats, which was restored to levels similar to those reached in CT after the administration of *Prodefen^®^ Plus*. Overall, the observed reduction of PKA activity in mesenteric arteries from MtS rats presumably induced a decrease in nNOS activation and, consequently, a diminished NO release. This inactivation of the PKA-nNOS-NO pathway was recovered by supplementation of *Prodefen^®^ Plus* for 4 weeks.

NO function can be also influenced by oxidative stress. The amount and the kind of dietary fatty acids can regulate complex intracellular signalling systems, thereby modulating cellular function and promoting a pro-oxidative microenvironment, which reduces NO bioavailability [5,62]. At the vascular level, an increase in superoxide anion metabolizes NO, forming peroxynitrite [27]. Hence, superoxide anion reduces the availability of NO and participates in the development of the vascular disturbances observed in MtS. Since different probiotic strains have been reported to potentiate several antioxidant routes [16,69], the alterations in NO observed in our experimental conditions might be due to modifications in vascular oxidative stress status. However, we found no differences among the different groups when measuring superoxide anion formation, allowing us to rule out a possible role of oxidative stress in the nitrergic modifications observed in the present study. We should point out that, although both smooth muscle and adventitial layers can be a source of superoxide anions, the endothelial layer, removed in the present study, also has a relevant role in producing these reactive oxygen species [70,71,72].

## 5. Conclusions

Overall, our data describe a beneficial effect of *Prodefen^®^ Plus* regarding the different metabolic associated to MtS. Additionally, *Prodefen^®^ Plus* improves different mechanisms implicated in the regulation of vascular tone, therefore ameliorating the hypertension associated to this condition. In conclusion, the commercially available formula *Prodefen^®^ Plus* could be considered an interesting non-pharmacological approach in MtS.

## Figures and Tables

**Table 1:** Body weight gain, food and water intake and lipid profile.

Parameters	CT	MtS	MtS-SYNB
Body weight increase ^1^ (%)	87.8 ± 2.1	111.3 ± 7.1 *	106.1 ± 9.1 ^+^
Epididymal adipose pad ^2^ (g/cm)	1.66 ± 0.08	3.30 ± 0.34 *	3.44 ± 0.33 ^+^
Visceral adipose pad ^2^ (g/cm)	1.39 ± 0.06	3.05 ± 0.35 *	3.54 ± 0.30 ^+^
Liver weight ^2^ (g/cm)	1.91 ± 0.07	2.01 ± 0.09	1.86 ± 0.09
Food intake ^3^ (g/day)	20.96 ± 0.47	16.48 ± 0.47 *	16.67 ± 0.86 ^+^
Calorie intake ^3^ (Kcal/day)	60.75 ± 1.34	75.80 ± 2.14 *	76.69 ± 3.93 ^+^
Water intake ^3^ (mL/day)	36.59 ± 2.08	35.91 ± 1.02	38.16 ± 0.76
TC ^4^ (mg/dL)	88.88 ± 5.07	81.8 ± 5.37	90.38 ± 5.93
TG ^4^ (mg/dL)	112.20 ± 55.80	156.00 ± 16.37 *	115.40 ± 6.89 ^#^
HDL ^4^ (mg/dL)	37.29 ± 2.32	30.37 ± 2.19	35.41 ± 2.57
LDL ^4^ (mg/dL)	20.61 ± 2.47	25.31 ± 3.53	31.89 ± 3.08

Notes: Data are expressed as mean ± S.E.M. Statistical analysis: One-way ANOVA followed by a Newman–Keuls *post-hoc* test. * *p* < 0.05 CT vs. MtS; # *p* < 0.05 MtS vs. MtS-SYNB; + *p* < 0.05 CT vs. MtS-SYNB. *n* = 8 animals per group. ^1^ Body weight increase is expressed as % of the initial body weight. ^2^ Respective weights were normalized to tibia length (in cm; CT: 5.25 ± 0.03; MtS: 5.26 ± 0.09; MtS-SYNB: 5.19 ± 0.09). ^3^ Food and water intake were measured every two days in the last 4 weeks of the experimental procedure coinciding with the administration of the synbiotic. ^4^ Serum total cholesterol (TC), triglycerides /TG), high density lipoproteins (HDL) and low density lipoproteins (LDL) were analysed at the end of the experimental period of 12 weeks. CT (control group), MtS (metabolic syndrome), MtS rats supplemented with *Prodefen® Plus* (MtS-SYNB).

**Table 2:** Systolic blood pressure.

	CT	MtS	MtS-SYNB
Week 0 ^1^	118.5 ± 1.8	110.9 ± 3.0 *	110.1 ± 2.7 ^+^
Week 8 ^2^	119.5 ± 2.4	141.1 ± 1.6 *	137.4 ± 1.0 ^+^
Week 12 ^3^	120.3 ± 3.7	145.3 ± 2.1 *	120.6 ± 1.6 ^#^

Notes: Results are expressed as mmHg (mean ± S.E.M). Statistical analysis: One-way ANOVA followed by a Newman–Keuls *post-hoc* test. * *p* < 0.05 CT vs. MtS; # *p* < 0.05 MtS vs. MtS-SYNB; + *p* < 0.05 CT vs. MtS-SYNB. *n* = eight animals per group.^1^ Beginning of procedure. ^2^ Eight weeks of dietary intervention. ^3^ Twelve weeks of dietary intervention (4 weeks of synbiotic supplementation in MtS + SYNB group).

**Table 3:** Nitric oxide and superoxide anion release in endothelium-denuded mesenteric rings.

	CT	MtS	MtS SYNB
NO release (A.F.U./mg tissue) ^1^	2518 ± 147	1065 ± 10 *	2498 ± 279 ^#^
Superoxide anion release(C.U./min/mg tissue) ^2^	55.26 ± 3.01	55.78 ± 8.13	55.74 ± 6.54

Notes: ^1^ Results are expressed as arbitrary fluorescence units (A.F.U.)/mg tissue. Mean ± S.E.M. Statistical analysis: One-way ANOVA followed by a Newman–Keuls *post-hoc* test. * *p* < 0.05 CT vs. MtS; # *p* < 0.05 MtS vs. MtS-SYNB. *n* = five to eight animals per group.^2^ Results are expressed as chemiluminescence units (C.U.)/minute/mg tissue. Mean ± S.E.M. *n* = five to eight animals per group.
